# Regional Brain Volume Changes Across Adulthood: A Multi-Cohort Study Using MRI Data

**DOI:** 10.3390/brainsci15101096

**Published:** 2025-10-11

**Authors:** Jae Hyuk Shim, Hyeon-Man Baek, Jung Hoon

**Affiliations:** 1 MTech Lab Co., Ltd., Room B1027, 119, Songdo Munhwa-ro, Yeonsu-gu, Incheon 21985, Republic of Korea; 2 Department of Health Sciences and Technology, GAIHST, Gachon University, Incheon 21999, Republic of Korea; 3 Mondrian AI, Room B1020, 119, Songdo Munhwa-ro, Yeonsu-gu, Incheon 21985, Republic of Korea; jhoon@mondrian.ai

**Keywords:** brain aging, MRI, volumetric analysis, neuroimaging, structural changes, neurodegeneration

## Abstract

**Background/Objectives:** Age-related structural changes in the human brain provide essential insights into cognitive aging and the onset of neurodegenerative diseases. This study aimed to comprehensively characterize age-related volumetric changes across multiple brain regions in a large, diverse, cognitively healthy cohort spanning adulthood (ages 21–90), integrating Korean, Information eXtraction from Images (IXI), and Alzheimer’s Disease Neuroimaging Initiative (ADNI) MRI datasets of cognitively healthy participants to characterize normative volumetric changes across adulthood using demographically diverse datasets. **Methods:** High resolution 3T T1-weighted MRI images from three distinct cohorts (totaling 1833 subjects) were processed through an optimized neuroimaging pipeline, combining advanced preprocessing with neural network-based segmentation. Volumetric data for 95 brain structures were segmented and analyzed across seven age bins (21–30 through 81–90). Pipeline reliability was validated against FreeSurfer using intraclass correlation coefficients (ICC) and coefficients of variation (CoV). Regression-based correction was used to correct for sex and cohort effects on brain region volume. Then, percentage change in each mean bilateral volumes of regions across the lifespan were computed to describe volumetric changes across life spans. **Results:** The segmentation pipeline demonstrated excellent agreement with FreeSurfer (mean ICC: 0.9965). Drastic volumetric expansions were observed in white matter hypointensities (122.6%), lateral ventricles (115.9%), and inferior lateral ventricles (116.8%). Moderate-to-notable shrinkage was found predominantly in the frontal lobe (pars triangularis: 21.5%), parietal lobe (inferior parietal: 20.4%), temporal lobe (transverse temporal: 21.6%), and cingulate cortex (caudal anterior cingulate: 16.1%). Minimal volume changes occurred in regions such as the insula (3.7%) and pallidum (2.6%). **Conclusions:** This study presents a comprehensive reference of normative regional brain volume changes across adulthood, highlighting substantial inter-regional variability. The findings can provide an essential foundation for differentiating normal aging patterns from early pathological alterations.

## 1. Introduction

Aging is a universal process marked by profound changes in brain structure, function, and connectivity [1,2,3]. These changes are central to understanding the neural basis of age-related cognitive decline and the heightened vulnerability to neurodegenerative diseases in older adults [4]. Across the lifespan, specific brain regions, such as the hippocampus, ventricles, and cortical lobes, exhibit dynamic alterations in volume and morphology, reflecting underlying neurobiological processes, including synaptic loss, myelin degradation, and cerebrovascular changes [5,6,7]. One particular method to detail such alterations is to measure the volumetric difference in brain regions from the subjects of various age groups through imaging tools such as MRI [8,9]. However, even recent large-scale normative imaging studies face challenges in assembling demographically diverse datasets across the full adult lifespan, often emphasizing midlife participants and predominantly single ethnicity populations [10,11,12].

To address such challenges, we investigate structural brain changes across a large, diverse cohort of subjects aged 20 to 90 years, leveraging high-resolution 3T MRI and neuroimaging tools capable of segmenting 95 brain structures. By integrating datasets from distinct sources, including a Korean population, a widely used European Information eXtraction from Images (IXI) dataset, and the predominantly Caucasian Alzheimer’s Disease Neuroimaging Initiative (ADNI) dataset, we aim to capture a comprehensive view of age-related trajectories across different demographics [13,14]. Importantly, this integration provides complementary normative references to existing single-cohort efforts by spanning early adulthood through late life and incorporating multiple ethnic groups, thereby supplementing underrepresented ethnicities and age ranges (e.g., pre-50 participants absent in ADNI).

In this study, we utilize the MTech Lab BrainMAP pipeline, an optimized combination of advanced neuroimaging tools for preprocessing and segmentation of MRI images. The pipeline integrates preprocessing techniques, such as denoising, intensity normalization, and spatial normalization, with FastSurfer, a neural network-based tool for a streamlined rapid brain structure segmentation orders of magnitude faster than traditional methods such as FreeSurfer [15,16]. Through the pipeline, we assess the combined means and standard deviation of brain region volumes, organized into age groups (21–30, 31–40, 41–50, 51–60, 61–70, 71–80, 81–90). Each brain region was then grouped by its respective lobe location (frontal, parietal, temporal, occipital, cingulate, other). To ensure the robustness of the BrainMAP pipeline, we compare its performance against FreeSurfer, the gold standard method for segmentation of brain structures from MRI images [17,18]. Using a sample of 20 subjects from each dataset, we evaluate the agreement between the two methods using metrics such as intraclass correlation coefficients (ICC), and coefficient of variation (CoV). We first corrected for sex and cohort related differences to reduce potential systematic variance, then calculated the percentage change in mean bilateral brain region volumes between age groups to detail patterns of volumetric change in aging. Additionally, we utilized polynomial regression analyses to describe non-monotonic volumetric changes in certain brain volumes. Through these analyses, we aim to contribute to the understanding of normative patterns of brain aging using an integrated dataset of diverse populations for a more generalized result.

## 2. Materials and Methods

### 2.1. Subjects

A total of 1833 subjects (M = 970, F = 863) spanning three different datasets were collected for this study. Mean age and demographics details per age group for each dataset are shown in Table 1.

#### 2.1.1. Korean Dataset

The dataset of 364 Korean subjects spanning ages from 21 to 90 (M = 220, F = 144) with no history of major disease was organized for this study. Three subjects above or equal to age 81 were excluded due to very small sample sizes. All participants were interviewed to ensure they did not have a history of any mental disorder, neurologic disease, psychiatric disorders, or other substance use disorders. The images were obtained from Gachon University School of Medicine using a 3T Philips Achieva Scanner with the same 32-channel heal coil and pulse sequence. The T1-weighted MPRAGE sequence was taken following closely with the ADNI protocol, which utilizes the following parameters: 6.8 ms TR, 3.1 ms TE, 256 mm FOV, 9° flip angle, 1 × 1 × 1.2 mm voxel size, 170 slices without gaps.

#### 2.1.2. IXI Dataset

The IXI dataset, which comprises 563 images of healthy subjects spanning ages from 20 to 86 were obtained from the publicly available IXI dataset (brain-development.org), which includes healthy subjects that were recruited to be scanned across multiple hospital sites in London, UK. For this study, we selected 563 T1-weighted images from unique individuals (312 females, 251 males). The IXI dataset contains data acquired at 1.5 T and 3 T field strengths across different scanner manufacturers (Philips Healthcare, Amsterdam, The Netherlands; GE Healthcare, Chicago, IL, USA); detailed acquisition parameters vary by site and are documented on the dataset’s website.

#### 2.1.3. ADNI Dataset

T1-weighted structural MRI data were obtained from the Alzheimer’s Disease Neuroimaging Initiative (ADNI) database (adni.loni.usc.edu), consisting of 909 subjects (M = 408, F = 501), spanning ages from 51 to 90. Each baseline 3D T1-weighted image was selected corresponding to the first available scan per individual to avoid redundancy from longitudinal follow-ups. Subjects included had a Clinical Dementia Rating (CDR) global score of ≤0.5 and a Mini-Mental State Examination (MMSE) score of ≥25, to ensure the subjects had normal cognition or very mild impairment. All images were acquired according to standardized ADNI MRI acquisition protocols, which are publicly available and designed to minimize inter-site variability across scanner platforms and field strengths [14].

Data used in the preparation of this article were obtained from the Alzheimer’s Disease Neuroimaging Initiative (ADNI) database (adni.loni.usc.edu). The ADNI was launched in 2003 as a public–private partnership, led by Principal Investigator Michael W. Weiner, MD. The original goal of ADNI was to test whether serial magnetic resonance imaging (MRI), positron emission tomography (PET), other biological markers, and clinical and neuropsychological assessment can be combined to measure the progression of mild cognitive impairment (MCI) and early Alzheimer’s disease (AD). The current goals include validating biomarkers for clinical trials, improving the generalizability of ADNI data by increasing diversity in the participant cohort, and providing data concerning the diagnosis and progression of Alzheimer’s disease to the scientific community. For up-to-date information, see adni.loni.usc.edu.

### 2.2. Image Processing

Each 3D T1w image from the three datasets were processed through the MTech Lab BrainMAP 1.0 fully automated image processing pipeline, which incorporates a series of preprocessing steps and 3D CNN-based FastSurfer to segment the whole brain into 95 anatomical and cortical structures in under a minute. The preprocessing steps involve normalizing the intensity of the image through N4 biasfield correction, conforming the orientation of the brain to the standard MNI152 space, then resampling the image to a 256 × 256 × 256 1 mm isotropic space. The preprocessed images are then fed to the FastSurfer CNN model that segments the image into 95 structures defined by the Desikan-Killiany-Tourville (DKT) atlas, as well as output the volumetric information of each segmented structure in mm^3^.

### 2.3. Statistical Analysis

To validate the segmentation accuracy and consistency of the BrainMAP pipeline, we compared its results against FreeSurfer 7.4, which has been recently enhanced with modules such as SynthSeg, SynthStrip, and SynthMorph for improved robustness and speed [19]. A subset of 20 subjects from each dataset (Korean, IXI, and ADNI) was processed using both pipelines. For this comparison, brain structures were grouped into six broader anatomical categories: subcortical nuclei, frontal lobe, parietal lobe, temporal lobe, occipital lobe, and limbic lobe. Within each lobe, 90 volumes from corresponding left and right hemisphere structures were combined to reduce interhemispheric redundancy. We computed mean volume differences between FreeSurfer and BrainMAP segmentations, standard deviation of differences, intraclass correlation coefficients (ICC), coefficients of variation (CoV), effect sizes (D), and coefficient of determination (R2). High intraclass correlation coefficients and low coefficients of variation would indicate strong agreement between the pipelines.

For comprehensive analysis and improved interpretability, the 95 anatomical regions (90 bilateral regions, 5 non-bilateral: 3rd and 4th ventricle, brain stem, white matter Hypointensities, cerebrospinal fluid) segmented by the BrainMAP pipeline were grouped into six categories based on neuroanatomical convention: subcortical (hippocampus, amygdala, caudate, accumbens, pallidum, putamen, thalamus, choroid plexus, ventral diencephalon (DC)), frontal lobe (caudal middle frontal, rostral middle frontal, superior frontal, lateral orbito frontal, medial orbito frontal, pars operacularis, pars orbitalis, pars triangularis, precentral), parietal lobe (inferior parietal, superior parietal, supra marginal, paracentral, postcentral, precuneus), temporal lobe (entorhinal, fusiform, insula, inferior temporal, middle temporal, superior temporal, transverse temporal, parahippocampal), occipital lobe (cuneus, lateral occipital, lingual, pericalcarine), cingulate lobe (caudal anterior cingulate, isthmus cingulate, posterior cingulate, rostral anterior cingulate), and other (cerebral white matter, cerebellum, cerebellum white matter, inferior lateral ventricles, 3rd and 4th ventricles, cerebrospinal fluid (CSF), brain stem, white matter (WM) hypointensities).

For each age group, the mean and standard deviation of these bilateral volumes were computed after excluding zero values. In addition, 95% confidence intervals (CI) were calculated using the standard error of the mean for each region’s volume distribution across age. Means of left and right hemisphere volumes were summed for each brain region to obtain combined bilateral measurements, then categorized into six separate tables (one for each anatomical category) to improve readability and to facilitate cross-regional comparison.

Regional volumes were adjusted for sex and cohort effects using linear regression models. For each brain region, volume was modeled as

Vi = β0 + β1(Sex_i_) + β2(Age_i_) + ∑k = 1K − 1γk1{Cohort_i_ = k} + ε_i,_

where Vi represents the observed volume for participant i, β0 is the intercept, β1, β2, and γk are regression coefficients, Sex_i_ was coded as Male = 1, Female = 0, Age_i_ represents chronological age as a continuous variable, 1{Cohort_i_ = k} is an indicator function equal to 1 if participant i belongs to cohort k and 0 otherwise, K represents the total number of datasets, and ε_i_ is the residual error term. Corrected volumes were computed by removing fitted sex and cohort contributions, preserving age-related variance and individual differences. We used the following equation to compute corrected volumes:

Vi_corrected_ = Vi − [β1(Sex_i_) + ∑k = 1K−1γk·1{Cohort_i_ = k}],

where Vi_corrected_ denotes the corrected volume for participant i and all other terms are as defined above. To verify removal of cohort and sex effects, we re-tested their residual associations on corrected volumes using the following linear model:

Vi_corrected_ = δ0 + δ1(Sex_i_) + ∑k = 1K − 1δk·1{Cohort_i_ = k} + η_i,_

where δ0 is the intercept, δ1 is the regression coefficient for sex, δk are regression coefficients for cohort k, η_i_ is the residual error term for participant i, and 1{Cohort_i_ = k} is an indicator function equal to 1 if participant i belongs to cohort k and 0 otherwise. Under successful correction, we expect δ1 ≈ 0 (no residual sex effect) and δk ≈ 0 for all k (no residual cohort effects). *p*-values for the post-correction coefficient tests were adjusted using the Benjamini–Hochberg false discovery rate across all regions (and across cohort levels where applicable).

To characterize non-linear age-related trajectories, we implemented a comprehensive polynomial regression analysis fitting linear and cubic models to each brain region. Non-monotonic patterns were identified using peak detection algorithms applied to smooth polynomial curves, with regions classified as U-shaped (local minimum in middle age), inverted-U (local maximum in middle age), or linear. The cubic model used to fit each brain region can be described by the following equation:

Vi = α0 + α1(Age_i_) + α2(Age_i_^2^) + α3(Age_i_^3^) + ε_i,_

where Vi represents mean volume for age group i, α0, α1, α2, and α3 are polynomial coefficients, and ε_i_ is the error term.

R^2^ improvement values over linear models were obtained for each pattern (U-shaped, inverted-U), and improvement values higher than 0.2 were considered more fit for that pattern than the conventional linear pattern. We then obtained three different measures to describe age-related change. Endpoint changes (% change) were used to compare the mean volumetric variability between the youngest age group (21–30) with the oldest age group (81–90). The endpoint changes were derived using the following equation:

[(V_old_ − V_young_)/((V_old_ + V_young_)/2)] × 100,

where V_young_ refers to the mean bilateral volume of the youngest age group (21–30 years), and V_old_ refers to the mean bilateral volume of the oldest age group (81–90).

To describe the largest percentage change between any two adjacent age groups, we obtained the maximum consecutive change using the following equation:

max{|[(V{i + 1} − V*i*)/((V{i + 1} + V*i*)/2)] × 100|} for i = 1, 2,…,7,

where Vi represents the mean volume for age group i, V{i + 1} represents the mean volume for the subsequent age group, and the age groups are indexed as follows: 1 = (21–30), 2 = (31–40), 3 = (41–50), 4 = (51–60), 5 = (61–70), 6 = (71–80), and 7 = (81–90 years). The maximum operator selects the largest absolute percentage change across all six possible consecutive age group pairs.

To describe the maximum variability across all age groups (comparing the minimum and maximum mean volumes across all age groups), we used the following equation:

[(V_max_ − V_min_)/((V_max_ +V_min_)/2)] × 100,

where V_max_ refers to the largest mean bilateral volume value out of all age groups (21–90 years), and V_min_ refers to the smallest mean bilateral volume value of all age groups (21–90 years).

All analyses were descriptive in nature. No formal hypothesis tests of aging effects were performed, as the aim of this study was to provide normative volumetric references rather than inferential comparisons.

## 3. Results

### 3.1. MTech Lab BrainMAP Pipeline Evaluation

We evaluated the agreement between the MTech Lab BrainMAP pipeline and FreeSurfer across seven lobes using multiple metrics, including intraclass correlation coefficient (ICC), coefficient of variation (CoV), reproducibility coefficient, and effect size. As shown in Table 2, the mean ICC was 0.9965 across lobes, indicating excellent volumetric agreement between the two segmentation pipelines. ICC values ranged from 0.9948 to 0.9976, with all lobes exceeding the conventional threshold of 0.90. The mean CoV was 5.55%, ranging from 4.43% to 6.87%. The subcortical and temporal lobes showed the lowest variability (CoV < 5%), while the limbic lobe had the highest CoV at 6.87%. The mean standard difference in volume estimates between BrainMAP and FreeSurfer was 518.99 mm^3^, with a maximum difference of 1135.47 mm^3^ observed in one lobe. Effect sizes (Cohen’s D) ranged from −0.07 to 0.46, with an average of 0.14, indicating mostly negligible to small differences. The mean reproducibility coefficient was 951.5 mm^3^. The mean coefficient of determination (R^2^) across lobes was 0.761, meaning that ~76% of the variance in FreeSurfer outputs could be explained by BrainMAP segmentations.

### 3.2. Regression-Based Correction for Sex and Cohort Confounds

Dual correction successfully eliminated sex and cohort confounds while preserving age-related variance. Prior to correction, sex differences were significant in 91/95 regions (95.8%, FDR < 0.05) with males showing larger volumes (mean Cohen’s d = 0.427, 95% CI: 0.401–0.453), and cohort effects were significant in 91/95 regions (95.8%). Post-correction, sex differences were reduced to significance in only 3/95 regions (FDR < 0.05 in 3rd Ventricle, left and right choroid plexus) and cohort effects were eliminated entirely (0/95 regions significant, 100% elimination rate). Mean absolute sex effect size decreased by 82.2% (d = 0.427 → 0.076, paired t_94_ = 23.7, *p* < 0.001). The correction preserved age-related variance (partial r^2^ = 0.31 ± 0.15) and individual differences (ICC = 0.94 ± 0.03).

### 3.3. Regional Brain Volume Trajectories Across the Lifespan

In total, 45 bilateral (90 pairs of left and right brain volumes combined to make 45 regions) and 5 non-bilateral brain volumes were summarized across 7 age bins (21–30 through 81–90 years) and grouped into six neuroanatomical lobes and an additional “Other Regions” category. The endpoint percent change in mean bilateral volume between the youngest (21–30) and oldest (81–90) age groups for each structure were computed and displayed in Table 3 to contextualize volumetric change. Each region’s trajectory was analyzed with respect to both magnitude and direction of change, allowing identification of regions with high susceptibility to age-related structural transformation.

#### 3.3.1. Subcortical Structures

**Figure 1:**
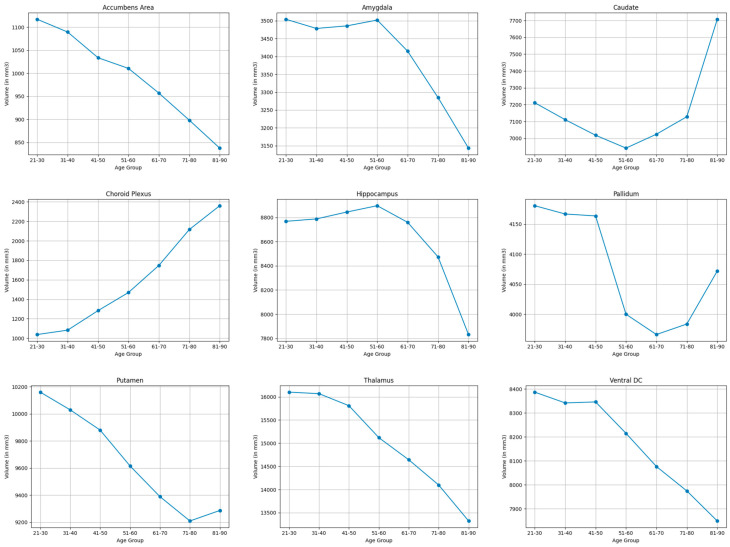
Mean volume (in mm^3^) of bilateral subcortical brain structures across the lifespan.

As shown in Table 3 and Table 4, and visualized in Figure 1, the choroid plexus showed the largest volumetric expansion (77.8%) out of the subcortical structures. Large shrinkage was observed in accumbens-area (28.6%), and moderate shrinkage was observed in the thalamus (18.9%), and the hippocampus (11.3%). More modest volume reductions were seen in the ventral DC (6.6%), putamen (9.0%), and amygdala (10.9%). The caudate (5.4%) and the pallidum (2.6%) demonstrated minimal volumetric change.

#### 3.3.2. Frontal Lobe

**Figure 2:**
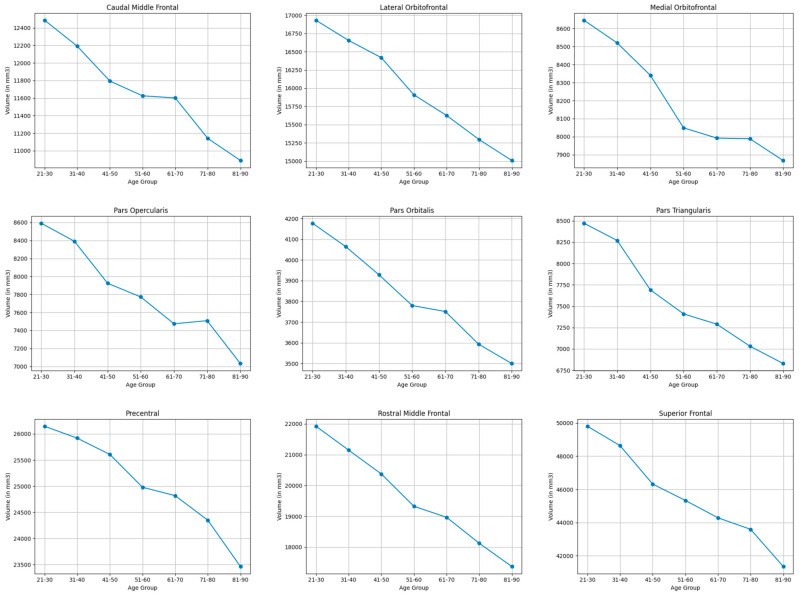
Mean volume (in mm^3^) of bilateral frontal lobe brain structures across the lifespan.

As shown in Table 3 and Table 5, and visualized in Figure 2, the frontal lobe regions showed notable shrinkage in the pars triangularis (21.5%), pars opercularis (20.0%), and the rostral middle frontal (23.2). Moderate volume reductions were seen in the precentral (10.8%), pars orbitalis (17.6%), caudal middle frontal (13.7%), superior frontal (18.6%), lateral orbitofrontal (12.0%), and medial orbitofrontal (9.4%).

#### 3.3.3. Parietal Lobe

**Figure 3:**
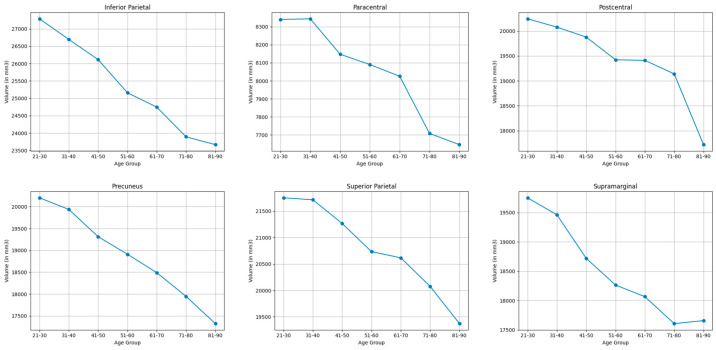
Mean volume (in mm^3^) of bilateral parietal lobe brain structures across the lifespan.

As shown in Table 3 and Table 6, and visualized in Figure 3, the parietal lobe demonstrated moderate shrinkages in the inferior parietal lobule (14.2%), superior parietal (11.6%), supramarginal (11.2%), precuneus (15.3%), postcentral (13.3%) and the paracentral (12.5%).

#### 3.3.4. Temporal Lobe

**Figure 4:**
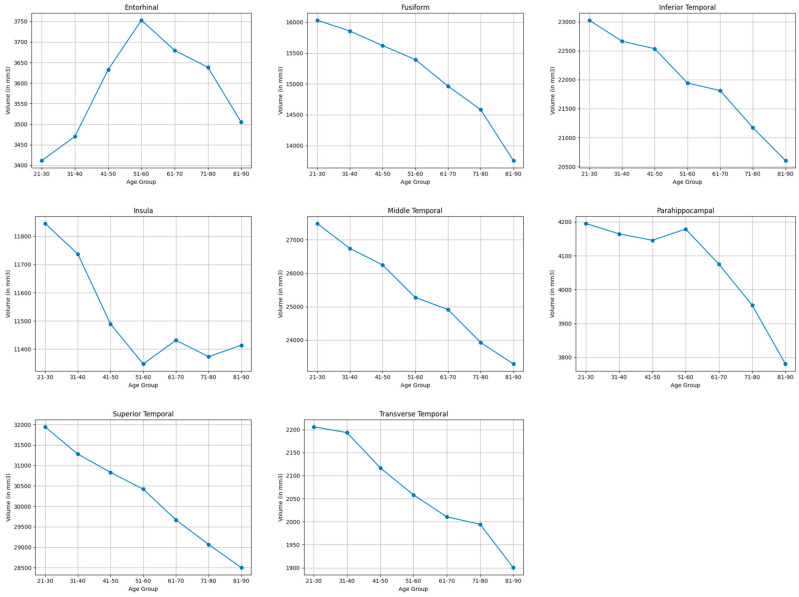
Mean volume (in mm^3^) of bilateral temporal lobe brain structures across the lifespan.

As shown in Table 3 and Table 7, and visualized in Figure 4, the temporal lobe showed moderate shrinkages in the transverse temporal (14.9%) middle temporal gyri (16.6%), superior temporal (11.4%), fusiform (15.3%), parahippocampal gyri (10.4%), inferior temporal (11.1%). The entorhinal showed minor increases (2.7%), with the insula showing minimal volume reduction (3.7%).

#### 3.3.5. Occipital Lobe

**Figure 5:**
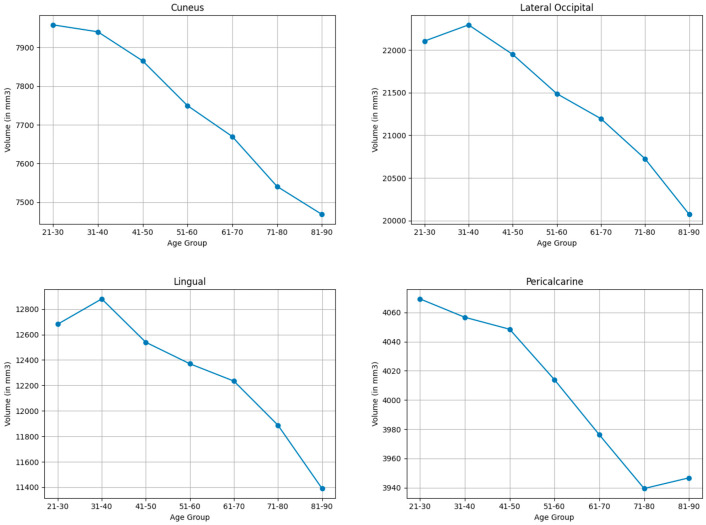
Mean volume (in mm^3^) of bilateral occipital lobe brain structures across the lifespan.

As shown in Table 3 and Table 8, and visualized in Figure 5, Volume changes in occipital regions were less pronounced than in frontal or parietal lobes. Occipital regions exhibited moderate volumetric changes in the lingual (10.7%) and the lateral occipital (9.6%). Minor shrinkages were found in the pericalcarine (2.5%) and the cuneus (6.4%).

#### 3.3.6. Cingulate Lobe

**Figure 6:**
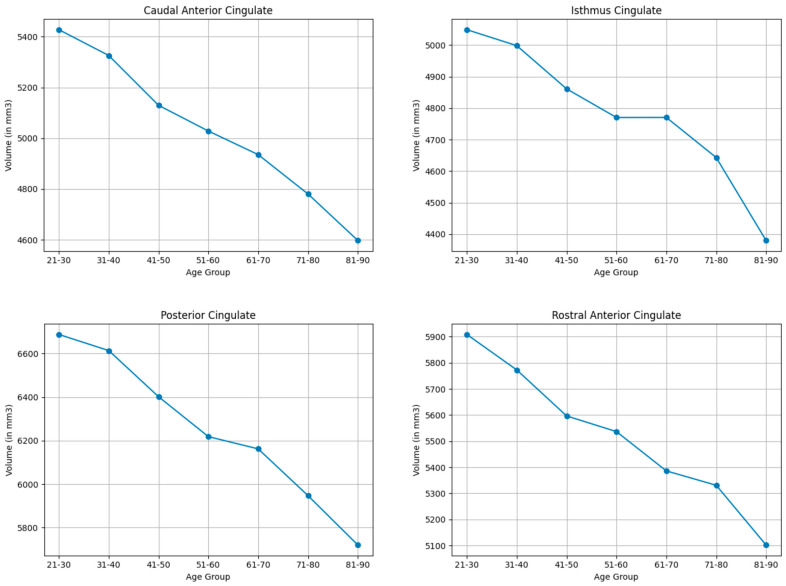
Mean volume (in mm^3^) of mean bilateral cingulate lobe brain structures across the lifespan.

As shown in Table 3 and Table 9, and visualized in Figure 6, the cingulate lobe regions demonstrated moderate shrinkage, most notably in the caudal anterior cingulate (16.6%) and posterior cingulate cortices (15.6%). Isthmus cingulate (14.2%) and rostral anterior cingulate (14.6%) also exhibited moderate volumetric reductions.

#### 3.3.7. Other Regions

**Figure 7:**
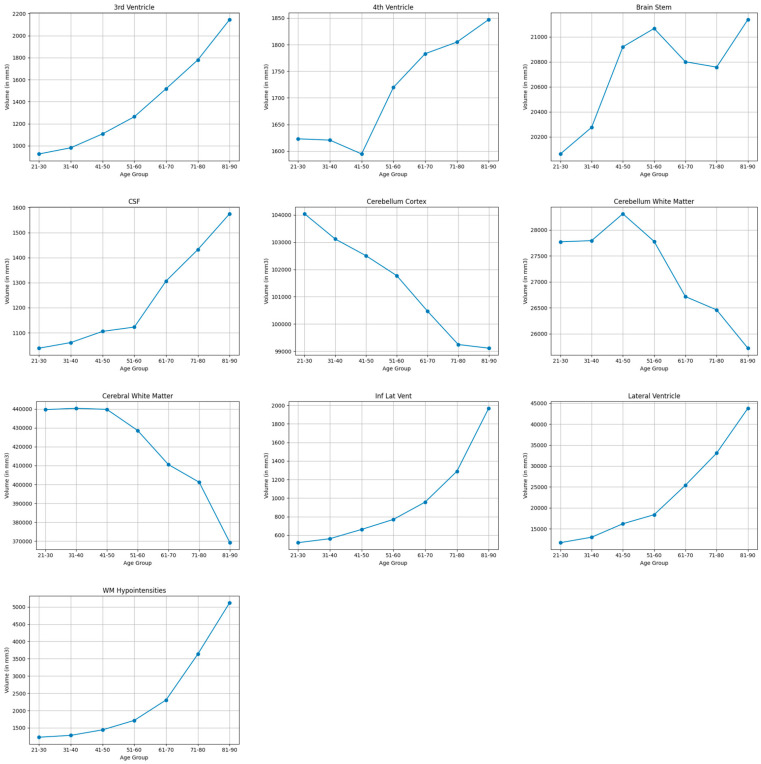
Mean volume (in mm^3^) of bilateral brain structures not assigned to cortical lobes across the lifespan.

As shown in Table 3 and Table 10, and visualized in Figure 7, regions not assigned to cortical lobes showed substantial volumetric expansions, particularly white matter hypointensities (122.6%), inferior lateral ventricles (116.8%), lateral ventricles (115.9%), and third ventricle (79.4%), cerebrospinal fluid (41.1%). Moderate volumetric increases occurred in the fourth ventricle (12.9%). The cerebral white matter showed moderate shrinkage (17.4%), while the cerebellar white matter (7.7%) and cerebellar cortex (4.9%) showed modest reductions. The brain stem demonstrated minimal volume increase (5.2%).

### 3.4. Non-Monotonic Changes

To describe non-monotonic age trajectories in brain volume changes, a comprehensive polynomial regression analysis was done to fit common non-linear patterns such as U-shaped, which describes a local minimum in middle age groups, and inverted-U, which describes a local maximum in middle age groups. As shown in Table 11, 3 regions exhibited U-shaped patterns (volume minimum in middle age): caudate (range change 10.4% vs. endpoint change 6.6%), pallidum (5.3% vs. −2.6%), insula (4.3% vs. −3.7%). 4 regions exhibited inverted-U patterns (volume maximum in middle age): entorhinal (9.5% vs. 2.7%), hippocampus (12.7% vs. −11.3%), amygdala (10.9% vs. −10.9%), and brain stem (5.2%).

## 4. Discussion

For this study, normative patterns of structural brain aging across broad and demographically diverse populations spanning ages 20 to 90 years were characterized using an integrated dataset composed of high resolution 3T MRI from three distinct cohorts involving Korean, European (IXI), and predominantly Caucasian (ADNI) populations. Initially, the MTech Lab BrainMAP segmentation and processing pipeline used to obtain the volumes of brain regions was validated for its robustness by calculating its agreement with FreeSurfer, the gold standard for segmentation of brain structures, on 20 subjects randomly selected from each population. The validation analysis demonstrated strong agreement between the BrainMAP pipeline and FreeSurfer pipeline, indicated by the high intraclass correlation coefficient (mean ICC = 0.9965) and low coefficients of variation (mean CoV = 5.55%) across all brain regions.

Subsequently after validation, the pipeline was used to process each 3T MRI in the integrated dataset for volumes of 95 brain regions. First, in order to reduce the effects of glaring issues in sex and cohort differences on brain volume, a regression-based was utilized to isolate age-related brain changes from demographic confounds by fitting separate models for each brain region. This approach modeled each region’s volume as a function of sex, age, and cohort membership, then removed only the fitted sex and cohort contributions while preserving age effects and individual differences. Before correction, sex differences were present in nearly all brain regions (91/95), with males typically showing larger volumes, and cohort effects were equally widespread, reflecting technical differences between datasets. The correction proved highly effective, reducing sex effect sizes by over 80% and completely eliminating cohort effects across all regions. Post-correction validation confirmed successful removal of unwanted effects, with sex differences remaining significant in only 3 regions and cohort effects eliminated entirely. The high preservation of individual differences (ICC = 0.94) and age-related variance (partial r^2^ = 0.31) indicated successful separation of demographic confounds from meaningful biological variability without artificially removing true age effects.

With the corrected data, we calculated endpoint changes using traditional young-to-old comparisons between the 21–30 and 81–90 age groups to quantify overall age-related volumetric changes. Calculation of percentage change in mean bilateral volumes across age groups showed that ventricular structures (inferior lateral ventricle, 116.8%; lateral ventricle, 115.9%; 3rd ventricle, 79.4%), choroid plexus (77.8%), CSF (41.1%) and white matter hypointensities (122.6%) exhibited the largest volumetric expansions. The frontal cortical regions demonstrated substantial volume reductions of up to 23.2%, whereas the parietal (15.3%), cingulate (16.6%), temporal (16.6%), and occipital (10.7%) showed modest shrinkages. Certain cortical and subcortical regions such as the pallidum (2.6%), insula (3.7%), pericalcarine (2.5%), amygdala (7.3%), and cerebellum cortex (4.9%), showed minimal volume shrinkages over the lifespan. Conversely, certain regions such as the caudate (6.6%), entorhinal (2.7%), brain stem (5.2%) showed modest volumetric increases.

As shown in Table 3, the most drastic volumetric expansion between age ranges was the white matter hypointensities (122.6%), an area that represent areas of signal abnormality on MRI associated with reduced tissue integrity, often due to cerebrovascular pathology such as ischemic injury, demyelination, and microvascular dysfunction [20,21,22,23]. The dramatic increase observed likely reflects progressive age-related vascular damage, potentially driven by chronic hypertension, arteriosclerosis, or small vessel disease, ultimately contributing to cognitive decline by disrupting neural connectivity and efficiency [20,24]. Regions demonstrating similar magnitude of volumetric expansion included the inferior lateral ventricles (116.8%), lateral ventricles (115.9%), third ventricle (79.4%), consistent with results of previous studies that have attributed the enlargement to generalized parenchymal atrophy and CSF redistribution [25,26,27]. Choroid plexus (77.8%) also showed significant enlargement with age, similar to previous studies that have attributed the correlation with compensatory hypertrophy to perfusion loss [28].

In aging populations, significant atrophy was also observed within frontal and parietal lobes integral to executive functions, spatial orientation, and sensory integration. The pars triangularis (21.5% reduction), rostral middle frontal gyrus (23.2%), and caudal middle frontal gyrus (13.7%) are critical for language processing, executive functioning, and working memory [29,30]. As such, their volumetric declines may contribute directly to cognitive deficits frequently observed in aging populations. Similarly, the inferior parietal lobule (14.2% reduction), involved in visuospatial processing and attention, exhibited atrophy, potentially underpinning age-related spatial and attentional deficits [31,32].

Additionally, aging populations demonstrated notable volumetric reductions in the temporal lobe structures associated with functions relating to auditory processing, including the transverse temporal gyrus (14.9% reduction) [33,34]. Such patterns could reflect deterioration of auditory processing speed and accuracy common in older adults [35,36]. Moderate declines in the cingulate cortex, particularly the caudal anterior cingulate (16.6%) and posterior cingulate (15.6%), suggest vulnerability in regions associated with emotion regulation and episodic memory retrieval, potentially contributing to subtle cognitive and emotional alterations observed in aging [37,38,39].

Conversely, certain regions displayed relative volumetric stability, particularly in the insula (3.7%), caudate nucleus (5.4%), pallidum (2.6%), and pericalcarine (2.5%), regions that support diverse functions, including interoceptive awareness, reward and motor control [40,41,42,43]. Their minimal volume change suggests preserved functionality or compensatory neuroplastic mechanisms mitigating typical aging effects, contributing to maintained cognitive and emotional functionality in older adults [44].

To identify potential non-linear age trajectories that might be missed by endpoint comparisons, we applied polynomial regression analysis and peak detection algorithms to classify regions as linear, U-shaped, or inverted-U patterns. While the majority of brain regions showed expected linear age-related changes, seven regions displayed clear non-monotonic trajectories with substantial improvements in model fit over linear approaches (R^2^ improvement range: 0.231–0.766). Three regions exhibited U-shaped patterns, characterized by volume minima in middle age: the caudate (6.6% endpoint change, 10.4% range change), pallidum (−2.6% endpoint change, 5.3% range change), and insula (−3.7% endpoint change, 4.3% range change). These basal ganglia and insular cortex regions may experience initial vulnerability during middle age due to metabolic stress or lifestyle factors, followed by structural stabilization through compensatory mechanisms or changes in tissue composition. In contrast, four regions showed inverted-U trajectories with volume maxima in middle age: the entorhinal cortex (2.7% endpoint change, 9.5% range change), hippocampus (−11.3% endpoint change, 12.7% range change), amygdala (−10.9% endpoint change, 10.9% range change), and brainstem (5.2% endpoint change, 5.2% range change). These limbic and memory-related structures may reach peak structural integrity in middle age before succumbing to age-related pathological processes, consistent with their known vulnerability to neurodegenerative changes and early involvement in conditions such as Alzheimer’s disease [3,5]. The presence of such patterns highlights the complexity of brain aging processes and suggests that linear models may not fully capture volumetric change dynamics in all brain structures [5,45].

This study has several limitations that should be acknowledged. First, the cross-sectional design limits causal inference and does not allow us to distinguish true age-related trajectories from potential cohort effects. Additionally, although preprocessing and regression-based adjustments were performed, residual scanner- or site-specific biases may remain. We attempted to mitigate these issues by statistically correcting for both sex and dataset effects, which substantially reduced systematic variance, but there were still regions that showed significant differences in three regions (3rd ventricle, left and right choroid plexus), and cannot fully exclude subtle harmonization differences. Second, potential confounding factors such as genetic background, environmental exposures, and socioeconomic status were not controlled for and may contribute to inter-cohort variability. Additionally, although the cohort consisted of cognitively healthy subjects, we cannot rule out potential diagnosed diseases or non-neuro related diseases that could affect the imaging of subjects. Third, sample sizes were uneven across age bins, particularly in the oldest groups. While we excluded the Korean 81–90 bin due to insufficient numbers (*n* = 3), results in the older bins of other cohorts should still be interpreted cautiously given possible small-sample noise. Fourth, although non-monotonic patterns were explicitly modeled and described, longitudinal data are ultimately required to confirm these trajectories and to distinguish age effects from generational influences. Fifth, volumetric variability was higher in certain regions, particularly the cerebellum, likely reflecting segmentation challenges in posterior fossa structures. Sixth, volumetric expansions in ventricles and white matter hypointensities, while consistent with prior aging literature and robust after dataset correction, may remain sensitive to acquisition differences and could partly reflect subclinical pathology in some participants. Finally, interpretations of functional or cognitive implications of volumetric changes remain speculative in the absence of behavioral data, and future multimodal studies are needed to link structural changes with functional outcomes.

## 5. Conclusions

In conclusion, the results of this study provide detailed insights into normative brain aging, highlighting substantial regional variability in structural changes. The results emphasize significant volumetric expansions in cerebrovascular-related regions and pronounced cortical atrophy in areas associated with higher-order cognitive functions, offering a critical reference for understanding aging processes and distinguishing pathological alterations.

## Figures and Tables

**Table 1:** Demographic information of cohorts.

Class	Korean Dataset			IXI				ADNI			
Age Group	Mean Age	Male	Female	Age Group	Mean Age	Male	Female	Age Group	Mean Age	Male	Female
21–30	24.8 ± 2.5	116 (65.5%)	61 (34.5%)	21–30	25.8 ± 2.7	45 (45.0%)	55 (55.0%)	21–30			
31–40	34.4 ± 3.3	24 (64.9%)	13 (35.1%)	31–40	34.7 ± 2.8	60 (60.6%)	39 (39.4%)	31–40			
41–50	45.6 ± 2.5	26 (66.7%)	13 (33.3%)	41–50	44.6 ± 2.8	41 (46.1%)	48 (53.9%)	41–50			
51–60	55.6 ± 3.1	23 (74.2%)	8 (25.8%)	51–60	55.8 ± 2.9	38 (38.4%)	61 (61.6%)	51–60	57.4 ± 2.0	12 (25.5%)	35 (74.5%)
61–70	65.7 ± 2.7	21 (40.4%)	31 (59.6%)	61–70	64.5 ± 3.0	47 (39.8%)	71 (60.2%)	61–70	66.5 ± 2.3	134 (40.1%)	200 (59.9%)
71–80	74.9 ± 2.6	8 (32.0%)	17 (68.0%)	71–80	73.3 ± 2.5	15 (30.6%)	34 (69.4%)	71–80	74.3 ± 2.8	195 (47.6%)	215 (52.4%)
81–90				81–90	83.1 ± 2.3	5 (62.5%)	3 (37.5%)	81–90	83.4 ± 2.5	67 (56.8%)	51 (43.2%)
Totals		218	143	Totals		251	312	Totals		501	408

Mean age expressed as mean ± standard deviation; (%) refers to percentage of subjects that are the specified gender among the age group.

**Table 2:** Key metrics evaluated when comparing FreeSurfer results with BrainMAP Pipeline.

Lobe	Mean FS	Mean BP	Std Diff	ICC	CoV (%)	Effect Size (D)	Reproducibility Coefficient	R^2^
Frontal lobe	96,197.14	96,794.65	432.70	0.997606	4.760	−0.102	786.60	0.774
Parietal lobe	78,422.02	75,781.82	490.23	0.997757	4.604	0.711	896.19	0.727
Temporal lobe	90,651.45	90,731.23	419.74	0.997655	4.705	0.007	765.04	0.804
Occipital lobe	45,375.36	44,142.82	411.82	0.995763	6.355	0.510	684.25	0.724
Limbic lobe	22,565.31	22,842.40	151.34	0.994188	6.876	−0.109	282.59	0.615
Cerebellum	131,172.50	126,812.46	1062.12	0.998271	3.923	1.052	1991.09	0.674

Mean FS refers to mean volume of FreeSurfer outputs in mm^3^; Mean BP refers to mean volume of BrainMAP pipeline outputs in mm^3^; Std Diff refers to standard deviation of differences; ICC refers to Intraclass correlation coefficient; CoV refers to coefficient of variance.

**Table 3:** Endpoint change in mean bilateral volumes across age groups.

Group	Region	Endpoint Change (%)	Group	Region	Endpoint Change (%)
Subcortical	Choroid Plexus	77.8	Parietal Lobe	Inferior Parietal	−14.2
	Thalamus	−18.9		Superior Parietal	−11.6
	Accumbens Area	−28.6		Supramarginal	−11.2
	Hippocampus	−11.3		Precuneus	−15.3
	Ventral DC	−6.6		Postcentral	−13.3
	Putamen	−9.0		Paracentral	−8.7
	Pallidum	−2.6	Temporal Lobe	Transverse Temporal	−14.9
	Amygdala	−10.9		Middle Temporal	−16.6
	Caudate	6.6		Superior Temporal	−11.4
Frontal Lobe	Pars Triangularis	−21.5		Fusiform	−15.3
	Pars Opercularis	−20.0		Parahippocampal	−10.4
	Precentral	−10.8		Inferior Temporal	−11.1
	Rostral Middle Frontal	−23.2		Entorhinal	2.7
	Pars Orbitalis	−17.6		Insula	−3.7
	Caudal Middle Frontal	−13.7	Occipital Lobe	Cuneus	−6.4
	Superior Frontal	−18.6		Lateral Occipital	−9.6
	Lateral Orbitofrontal	−12.0		Lingual	−10.7
	Medial Orbitofrontal	−9.4		Pericalcarine	−2.5
Cingulate Lobe	Caudal Anterior Cingulate	−16.6	Other Regions	WM Hypointensities	122.6
	Posterior Cingulate	−15.6		Inf Lat Vent	116.8
	Isthmus Cingulate	−14.2		Lateral Ventricle	115.9
	Rostral Anterior Cingulate	−14.6		3rd Ventricle	79.4
				CSF	41.1
				4th Ventricle	12.9
				Cerebral White Matter	−17.4
				Cerebellum White Matter	−7.7
				Cerebellum Cortex	−4.9
				Brain Stem	5.2

**Table 4:** Mean values of bilateral subcortical structures in mm^3^ per age group.

Age Group	Thalamus	Caudate	Putamen	Pallidum	Hippocampus
21–30	16,102.0 ± 688.1	7211.5 ± 456.6	10,160.6 ± 561.8	4180.8 ± 230.3	8768.0 ± 398.0
31–40	16,068.8 ± 767.5	7111.0 ± 513.2	10,029.8 ± 602.7	4167.0 ± 232.5	8787.7 ± 414.2
41–50	15,809.4 ± 738.8	7018.5 ± 423.7	9880.5 ± 612.8	4163.8 ± 294.3	8844.8 ± 479.3
51–60	15,120.0 ± 859.2	6942.1 ± 430.6	9615.7 ± 623.0	4000.1 ± 288.8	8897.0 ± 483.3
61–70	14,642.0 ± 788.4	7024.6 ± 509.3	9389.2 ± 556.9	3966.3 ± 240.2	8759.4 ± 473.0
71–80	14,097.8 ± 723.3	7128.5 ± 560.4	9210.0 ± 674.4	3983.8 ± 331.4	8473.5 ± 485.9
81–90	13,322.4 ± 779.7	7707.0 ± 693.6	9287.4 ± 768.4	4072.0 ± 393.8	7831.8 ± 835.4
	**Amygdala**	**Accumbens-Area**	**VentralDC**	**Choroid-Plexus**	
21–30	3504.3 ± 195.0	1117.3 ± 90.9	8386.9 ± 386.2	1038.0 ± 219.2	
31–40	3478.6 ± 197.2	1089.6 ± 93.0	8341.9 ± 435.5	1083.0 ± 231.3	
41–50	3485.8 ± 186.6	1033.8 ± 89.7	8346.0 ± 491.1	1283.5 ± 294.4	
51–60	3502.2 ± 214.2	1010.6 ± 96.1	8214.9 ± 495.3	1469.5 ± 335.8	
61–70	3414.9 ± 225.4	957.0 ± 90.7	8076.4 ± 440.8	1747.6 ± 335.4	
71–80	3284.8 ± 255.1	898.2 ± 101.5	7974.4 ± 422.7	2115.8 ± 373.0	
81–90	3143.3 ± 295.2	837.6 ± 127.4	7849.0 ± 477.4	2360.5 ± 162.6	

Values expressed as mean ± standard deviation.

**Table 5:** Mean values of bilateral frontal lobe structures in mm^3^ per age group.

Age Group	Caudal Middle Frontal	Rostral Middle Frontal	Superior Frontal	Lateral Orbitofrontal	Medial Orbitofrontal
21–30	12,486.5 ± 1289.9	21,922.7 ± 1836.4	49,810.0 ± 2707.4	16,931.2 ± 847.9	8646.1 ± 518.0
31–40	12,190.6 ± 1250.7	21,147.6 ± 1830.5	48,641.8 ± 2775.8	16,654.9 ± 808.6	8520.1 ± 482.5
41–50	11,793.3 ± 1075.4	20,376.7 ± 1771.8	46,321.7 ± 2214.7	16,418.7 ± 869.3	8340.2 ± 505.5
51–60	11,625.6 ± 1252.8	19,325.9 ± 1677.7	45,332.9 ± 2560.8	15,908.3 ± 966.5	8048.9 ± 487.6
61–70	11,601.5 ± 1138.3	18,969.4 ± 1484.8	44,283.4 ± 2330.1	15,625.4 ± 834.8	7992.3 ± 519.8
71–80	11,139.7 ± 1170.7	18,126.7 ± 1475.3	43,587.9 ± 2406.9	15,294.0 ± 857.0	7988.3 ± 534.5
81–90	10,887.9 ± 935.7	17,371.2 ± 1432.5	41,341.8 ± 2261.1	15,007.5 ± 916.1	7867.5 ± 552.3
	**Pars Opercularis**	**Pars Orbitalis**	**Pars Triangularis**	**Precentral**	
21–30	8591.5 ± 912.9	4177.4 ± 410.2	8471.4 ± 975.3	26,147.5 ± 1431.3	
31–40	8391.1 ± 834.6	4064.4 ± 387.3	8269.6 ± 937.3	25,919.3 ± 1369.7	
41–50	7924.6 ± 747.6	3928.5 ± 409.6	7691.0 ± 814.3	25,607.4 ± 1387.9	
51–60	7772.4 ± 820.8	3779.9 ± 425.1	7410.3 ± 823.6	24,979.6 ± 1552.2	
61–70	7474.0 ± 720.3	3751.0 ± 368.7	7289.9 ± 791.9	24,818.3 ± 1374.3	
71–80	7508.6 ± 787.8	3593.5 ± 395.4	7032.1 ± 795.4	24,348.9 ± 1496.9	
81–90	7032.7 ± 796.8	3499.9 ± 333.1	6829.2 ± 958.6	23,461.5 ± 1441.3	

Values expressed as mean ± standard deviation.

**Table 6:** Mean values of bilateral parietal lobe structures in mm^3^ per age group.

Age Group	Inferior Parietal	Superior Parietal	Supramarginal	Paracentral	Postcentral	Precuneus
21–30	27,287.2 ± 1952.5	21,753.5 ± 1611.4	19,751.3 ± 1637.2	8340.5 ± 623.4	20,243.6 ± 1400.3	20,202.4 ± 1240.8
31–40	26,695.6 ± 1992.0	21,716.2 ± 1719.9	19,461.2 ± 1526.4	8343.9 ± 584.7	20,076.8 ± 1296.3	19,936.1 ± 1234.8
41–50	26,114.9 ± 1944.4	21,267.4 ± 1564.3	18,719.0 ± 1430.9	8147.6 ± 554.2	19,877.4 ± 1318.6	19,312.6 ± 1273.6
51–60	25,159.9 ± 1825.0	20,734.9 ± 1808.0	18,264.1 ± 1567.7	8090.1 ± 650.3	19,422.3 ± 1384.0	18,910.9 ± 1298.3
61–70	24,746.4 ± 1842.8	20,616.4 ± 1590.7	18,066.0 ± 1288.4	8025.3 ± 568.7	19,411.2 ± 1211.2	18,489.6 ± 1219.5
71–80	23,891.7 ± 1691.4	20,077.9 ± 1558.4	17,607.1 ± 1387.9	7708.5 ± 636.0	19,139.8 ± 1454.1	17,945.4 ± 1173.6
81–90	23,666.2 ± 2030.8	19,371.4 ± 1470.0	17,657.9 ± 1630.6	7646.3 ± 573.3	17,722.2 ± 1240.4	17,322.5 ± 1186.0

Values expressed as mean ± standard deviation.

**Table 7:** Mean values of bilateral temporal lobe structures in mm^3^ per age group.

Age Group	Entorhinal	Fusiform	Inferior Temporal	Middle Temporal
21–30	3411.4 ± 322.7	16,032.7 ± 1086.1	23,026.8 ± 1647.3	27,488.6 ± 1821.6
31–40	3470.3 ± 298.7	15,856.8 ± 1125.0	22,664.4 ± 1548.8	26,742.5 ± 1861.2
41–50	3632.6 ± 340.9	15,620.9 ± 1261.7	22,535.3 ± 1482.2	26,247.9 ± 1761.3
51–60	3752.8 ± 361.5	15,395.1 ± 1214.5	21,944.0 ± 1622.5	25,274.7 ± 1792.8
61–70	3679.4 ± 339.2	14,963.8 ± 1086.5	21,812.1 ± 1579.1	24,914.6 ± 1756.3
71–80	3638.2 ± 373.7	14,581.6 ± 1347.1	21,174.2 ± 1495.5	23,925.7 ± 1578.1
81–90	3505.2 ± 387.2	13,755.0 ± 1240.8	20,603.2 ± 1631.4	23,286.0 ± 2112.2
	**Superior temporal**	**Transverse temporal**	**Parahippocampal**	**Insula**
21–30	31,945.2 ± 1668.5	2205.9 ± 244.0	4195.2 ± 324.6	11,845.5 ± 644.0
31–40	31,279.1 ± 1568.6	2193.3 ± 232.9	4164.5 ± 338.7	11,737.2 ± 582.7
41–50	30,832.1 ± 1618.9	2116.8 ± 243.1	4145.6 ± 349.0	11,488.9 ± 644.2
51–60	30,421.5 ± 1558.1	2058.1 ± 232.7	4178.7 ± 332.6	11,347.3 ± 629.4
61–70	29,670.2 ± 1551.5	2010.6 ± 221.4	4075.0 ± 342.4	11,431.3 ± 634.0
71–80	29,069.7 ± 1712.7	1994.5 ± 253.8	3954.4 ± 379.8	11,373.1 ± 755.1
81–90	28,501.4 ± 1487.0	1900.3 ± 187.5	3780.6 ± 247.1	11,413.8 ± 1118.6

Values expressed as mean ± standard deviation.

**Table 8:** Mean values of bilateral occipital lobe structures in mm^3^ per age group.

Age Group	Cuneus	Lateral Occipital	Lingual	Pericalcarine
21–30	7958.4 ± 765.0	22,105.1 ± 1766.5	12,681.7 ± 1089.6	4069.3 ± 565.2
31–40	7940.5 ± 741.5	22,295.0 ± 1706.9	12,880.7 ± 1005.3	4056.7 ± 493.3
41–50	7865.3 ± 748.9	21,948.6 ± 1654.1	12,539.5 ± 1056.1	4048.5 ± 495.2
51–60	7749.6 ± 803.3	21,488.2 ± 1513.2	12,370.2 ± 1056.1	4013.9 ± 566.5
61–70	7669.7 ± 744.0	21,195.1 ± 1676.5	12,233.9 ± 1130.4	3976.2 ± 525.9
71–80	7540.5 ± 663.6	20,724.8 ± 1676.3	11,887.0 ± 1029.4	3939.4 ± 525.0
81–90	7468.2 ± 526.6	20,073.3 ± 1273.8	11,389.9 ± 512.2	3946.7 ± 292.7

Values expressed as mean ± standard deviation.

**Table 9:** Mean values of bilateral cingulate lobe structures in mm^3^ per age group.

Age Group	Caudal Anterior Cingulate	Isthmus Cingulate	Posterior Cingulate	Rostral Anterior Cingulate
21–30	5427.4 ± 594.8	5049.1 ± 417.7	6687.3 ± 511.6	5908.0 ± 630.6
31–40	5325.9 ± 581.9	4998.5 ± 412.2	6613.4 ± 519.0	5772.6 ± 605.3
41–50	5129.6 ± 551.3	4861.3 ± 417.9	6401.5 ± 495.1	5596.6 ± 651.2
51–60	5028.3 ± 598.6	4770.4 ± 411.7	6218.5 ± 548.7	5536.7 ± 606.6
61–70	4935.4 ± 567.2	4770.6 ± 411.2	6162.3 ± 515.5	5386.2 ± 536.7
71–80	4780.5 ± 530.3	4643.4 ± 361.8	5947.1 ± 546.6	5331.2 ± 539.6
81–90	4597.4 ± 488.7	4380.2 ± 303.4	5720.0 ± 505.6	5102.2 ± 562.1

Values expressed as mean ± standard deviation.

**Table 10:** Mean values of bilateral structures not assigned to cortical lobes in mm^3^ per age group.

Age Group	3rd Ventricle	4th Ventricle	Brain Stem	CSF	Cerebellum Cortex
21–30	925.8 ± 233.6	1623.1 ± 461.3	20,063.7 ± 1481.9	1038.2 ± 210.1	104,041.9 ± 5217.2
31–40	981.8 ± 246.3	1620.7 ± 471.1	20,276.2 ± 1682.3	1060.7 ± 186.9	103,116.1 ± 5434.5
41–50	1108.1 ± 286.4	1594.7 ± 454.9	20,919.9 ± 1648.7	1105.4 ± 218.7	102,503.6 ± 5056.1
51–60	1263.6 ± 379.2	1719.7 ± 512.6	21,066.2 ± 1758.4	1122.5 ± 254.0	101,774.1 ± 6110.6
61–70	1517.8 ± 512.5	1783.0 ± 492.2	20,800.3 ± 1784.5	1306.4 ± 258.6	100,470.9 ± 5205.3
71–80	1779.5 ± 484.5	1804.8 ± 498.5	20,757.4 ± 1625.8	1432.0 ± 310.3	99,246.9 ± 5330.8
81–90	2145.6 ± 408.1	1847.1 ± 654.0	21,137.5 ± 1663.5	1574.8 ± 347.1	99,111.7 ± 8363.4
	**Cerebellum White Matter**	**Cerebral White Matter**	**Inf Lat Vent**	**Lateral Ventricle**	**WM Hypointensities**
21–30	27,770.2 ± 1743.4	439,677.3 ± 32,474.0	519.2 ± 192.9	11,665.7 ± 4590.5	1228.9 ± 305.5
31–40	27,792.8 ± 1964.6	440,415.1 ± 33,528.7	561.7 ± 198.5	12,965.3 ± 4428.5	1282.3 ± 296.6
41–50	28,310.0 ± 2200.3	439,806.4 ± 35,521.2	661.9 ± 213.3	16,186.8 ± 5277.9	1441.9 ± 392.5
51–60	27,778.5 ± 2124.5	428,620.7 ± 36,962.6	769.6 ± 259.6	18,347.8 ± 6583.2	1715.7 ± 744.2
61–70	26,715.3 ± 1880.9	410,573.7 ± 34,680.2	956.6 ± 415.8	25,387.0 ± 8283.9	2306.2 ± 1525.2
71–80	26,458.7 ± 2042.4	401,222.6 ± 31,738.3	1288.8 ± 477.1	33,083.3 ± 10,544.6	3634.4 ± 3037.0
81–90	25,720.5 ± 2719.7	369,167.5 ± 27,960.6	1968.0 ± 568.8	43,811.9 ± 11,977.9	5121.1 ± 2555.4

Values expressed as mean ± standard deviation; Inf-Lat-Vent refers to inferior lateral ventricles; WM-hypointensities refers to white matter Hypointensities.

**Table 11:** Brain Regions with Non-Monotonic Age Trajectories.

Region	Pattern	R^2^ Improvement †	Endpoint Change (%)	Range Change (%)	Max Consecutive Change (%)
Caudate	U-shaped	0.766	6.6	10.4	7.8
Entorhinal	Inverted-U	0.751	2.7	9.5	4.6
Hippocampus	Inverted-U	0.471	−11.3	12.7	7.9
Pallidum	U-shaped	0.402	−2.6	5.3	4
Insula	U-shaped	0.244	−3.7	4.3	2.1
Amygdala	Inverted-U	0.24	−10.9	10.9	4.4
Brain Stem	Inverted-U	0.231	5.2	5.2	3.1

Pattern: Shape of age trajectory (U-shaped = minimum in middle age; Inverted-U = maximum in middle age); R^2^ Improvement †: Improvement in explained variance over linear model (higher values = more non-linear); Endpoint Change: Traditional young-old comparison (21–30 Vs. 81–90 age groups); Range Change: Maximum variability across all age groups (captures middle-age effects); Max Consecutive: Largest change between adjacent age groups (identifies steepest transitions).

## Data Availability

The original contributions presented in this study are included in the article. Further inquiries can be directed to the corresponding author.

## Abbreviations

The following abbreviations are used in this manuscript:

ADNI	Alzheimer’s Disease Neuroimaging Initiative
BP	BrainMAP Pipeline
CDR	Clinical Dementia Rating
CI	Confidence Inteval
CoV	Coefficient of Variation
DC	Diencephalon
FS	FreeSurfer
ICC	Intraclass Correlation Coefficient
IXI	Information eXtraction from Images
MMSE	Mini-Mental State Examination
MRI	Magnetic Resonance Imaging
WM	White Matter
